# Suitability of Different Variants of Polyethylene Glycol Impregnation for the Dimensional Stabilization of Oak Wood

**DOI:** 10.3390/polym10010081

**Published:** 2018-01-16

**Authors:** Tillmann Meints, Christan Hansmann, Wolfgang Gindl-Altmutter

**Affiliations:** 1 Wood K Plus—Competence Centre for Wood Composites and Wood Chemistry, Altenberger Stasse 69, 4040 Linz, Austria; c.hansmann@kplus-wood.at; 2 Department of Material Science and Process Engineering, Institute of Wood Technology and Renewable Materials, University of Natural Resources and Life Science (BOKU), Konrad Lorenzstrasse 24, 3430 Tulln, Austria; wolfgang.gindl-altmutter@boku.ac.at

**Keywords:** silane, polyethylene glycol (PEG), oak wood, impregnation, anti-shrinkage efficiency (ASE), wood modification

## Abstract

The common method to impregnate wood with polyethylene glycol (PEG) is to store the samples for several weeks in aqueous PEG-solution, allowing for diffusion of PEG into the wood. As this method is poorly suited for industrial application, an alternative approach based on vacuum-pressure treatment is evaluated in the present study. Using European oak wood and three variants of PEG, including silane-functionalized PEG, impregnation experiments at different PEG concentrations were performed. Significant uptake of PEG resulted in clearly altered wood-water relations and improved dimensional stability of oak wood. These results are discussed in terms of stability in humid and aqueous environments, and in terms of effects of the anatomy of oak wood on differences in dimensional stabilization observed along the radial and tangential anatomical directions, respectively. While both of the PEG variants perform better with an anti-shrinkage efficiency of up to 80%, the PEG-silane variant performs less effectively in this respect; however PEG-silane is clearly predominant in case of water extraction.

## 1. Introduction

Wood as a natural material features several beneficial properties such as good mechanical performance at comparably low weight, biodegradability, and renewability. On the downside, lack of dimensional stability diminishes the competitiveness of wood as an engineering material in selected application fields. This shortcoming of wood can be compensated for to a certain extent by appropriate choice of wood species for specific applications [1]. However, wood has clear limitations when it comes to more specialized applications and extreme environments. The dimensional instability of wood in environments of variable humidity is a major issue in the application of wood in critical climate conditions (e.g., window frames, exterior cladding, solid wood flooring, etc.). The pronounced sensitivity of wood to changing water content can be reduced by wood modification. Wood modification techniques can be grouped in active and passive approaches [2]. Active wood modification involves the chemical alteration of wood structure, either by means of derivatization or cross-linking, or by thermal modification. Passive wood modification features filling of cavities and/or cell walls with modification agents without any chemical reaction with the cell wall taking place. Passive modification usually is not that durable, and prone to leaching of the modification agent. An extensive overview of different modification approaches is given by Hill [3].

Emulsions of different waxes may be used to impregnate wood in order to endow it with improved surface properties, fungal resistance, and dimensional stability. A state of the art overview of such treatments is given by Kocaefe, et al. [4]. The treatment of wood with wax emulsions for the improvement of dimensional stability using vacuum pressure impregnation technology was investigated [5]. It was found that impregnation with different wax emulsions can cause an increase of wood mass of up to 10% for spruce and up to 16% for beech. Scholz, et al. [6] investigated the ability of impregnation of pine and beech with different waxes. Rays in pine wood seem to play an important role in terms of pathways for the impregnation agent. Due to their hydrophobicity, waxes tend to accumulate in cell cavities without penetrating into the cell wall itself.

Reactive impregnation agents such as melamine formaldehyde resins (MF) or phenol formaldehyde resins (PF) provide more efficient dimensional stabilization compared to wax emulsions. These resins are of sufficiently polar character and low molecular weight to enter the wood cell wall by diffusion, where they form an interpenetrating network upon curing, which greatly improves dimensional stability [7,8]. A mass increase of 25% was achieved with aqueous impregnation of Norway spruce [9]. With MF-modified common bur-flower (*Anthocephalus cadamba* Miq.), a mass increase of up to 34.1%, resulting in 68.2% reduced shrinkage upon drying was found [10]. Besides these formaldehyde-based resins, also non-aqueous mono/oligomer systems such as styrene or styrene-methylmetacrylate mixtures may be employed for impregnation of wood and subsequent in situ polymerization [11]. Finally, silicon-based aqueous wood modification agents dispose of a huge potential in wood modification due to numerous functionalization available [12,13,14]. Since they are capable to enter the wood cell wall, they achieve a certain bulking effect. Depending on the functionality, different wood properties can be obtained [15].

In contrast to waxes and some reactive modification agents, polyethylene glycol (PEG) is highly hydrophilic and non-reactive. On the one hand, it is easily dissolved in water, but on the other hand it is easily washed out again from impregnated wood in contact with water. The dimension-stabilizing properties of PEG-impregnated wood are well known. Stamm and Hansen [16] started to investigate possibilities of dimensional stabilization of wood in the 1930s. In the 1950–1970s, special focus was put on PEG [17,18,19,20,21,22]. The common method to impregnate wood with PEG is to store the samples days, weeks, or months, depending on the specimen dimensions, submerged in the PEG-water-solution and enable the PEG to diffuse into the wood [19,20]. Schneider [21] did extensive research on PEG treatment of pine and beech wood. He found good dimension stabilization of up to 90%. Higher values were possible, but with PEG content the wood became “moist” on the surface due to adsorbed water. Schneider [21] describes the bulking effect of PEG in beech and pine wood as a blocking effect of PEG that diffuses into the cell wall during treatment and additionally during drying/conditioning. Stamm [18] found increased shrinkage reduction, when the moisture content of wood prior to treatment was equal to or more than 40%. To ensure optimal PEG diffusion, it is recommended to use green or water-saturated wood. Additionally, after the impregnation, a homogenization step for PEG is recommended, to enable the PEG to diffuse deeper into the cell wall [18]. These findings were confirmed by Tanaka, et al. [23] by means of swelling investigations during conditioning phases after impregnation. Stamm [18] investigated the suitability of different molecular weight of PEG for efficient impregnation of spruce wood. It was found that the PEG uptake was optimal for a molecular weight of 550–1000, while the maximum bulking was achieved using PEG 350–550, indicating that lower molecular weight molecules seem to better penetrate the cell wall, compared to high molecular weight PEG.

At present, PEG treatment is mainly applied in the conservation of waterlogged archaeological wood. Since the archaeological objects are very fragile, it is aimed at replacing all the water in the cell wall by PEG of different molecular weight. All these treatments take place at atmospheric pressure. Prominent cases are for example the PEG-preserved “Bremen cog” [24,25] and the Swedish warship “Vasa” [26]. Even though the method works well in general, a disadvantage was revealed recently: due to iron catalyzed chemical wood degradation [27], the strength of the PEG-treated wooden material is negatively affected. Bjurhager, et al. [26] studied the mechanical properties of PEG 600 impregnated oak wood on small specimens (3 weeks treatment). They found only a slight reduction in axial tensile modulus and strength, but detected up to 50% lower compressive modulus and yield strength in radial direction. The observation is explained by changed micro fibril angle, caused by the swelling of the specimens, especially in the wood rays [28].

For highest cell wall loadings with PEG, the drying or conditioning phase after impregnation also is of importance besides impregnation as such. Slow drying after impregnation enables continued and deeper diffusion of PEG into the cell wall. The driving factor here is the fact that PEG concentration in the cell cavities increases with drying, i.e., evaporation of the PEG-solvent water, which leads to a PEG concentration gradient between cell cavity and cell wall. Tanaka, et al. [23] did intensive studies on that topic using PEG 1500 impregnated Hinoki wood (*Chamaecyparis obtuse*). Notably, Jeremic, et al. [29] show that a vacuum-driven impregnation of PEG 1000-PEG 4000 (dissolved in toluene) for only 15 min can be sufficient to achieve satisfying loadings of pine wood. Same as in earlier studies, higher PEG uptake was found when using water-saturated or green wood, compared to treatment of dried wood [18,21,30]. The resulting reduction in dimensional change not necessarily needs to be equal in the radial and anatomical directions of impregnated wood. Schneider [21] found for beech and pine a higher dimensional stabilization in radial direction compared to tangential direction.

In the present study, a new improved approach to PEG impregnation is followed by combining the beneficial features of PEG with the advantages of silanes for wood modification. Silanes are capable of adsorbing to the wood surface [31] or cellulosic fibers [32]. Additionally, self-condensation or covalent attachment to lignocellulosic surfaces may take place [13], which is advantageous with regard to minimizing leaching of impregnation reagent in aqueous environment. Impregnation experiments are thus conducted with PEG and with silane-functionalized PEG, and the resulting wood property improvements are comprehensively characterized.

## 2. Materials and Methods

### 2.1. Wood Material and Treatment

**Figure 1:**
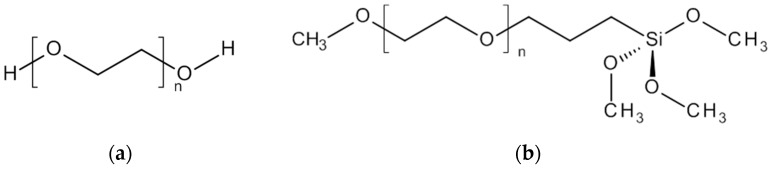
PEG 400 with *n* ≈ 8.5 and PEG 1000 with *n* ≈ 22 (**a**); PEG-silane with *n* ≈ 7.5 (**b**).

One hundred European oak wood specimens (*Quercus* spec.) were cut to dimensions of 25 × 24 × 10 mm^3^ (radial × tangential × longitudinal). All specimens were dried at 103 °C for 48 h to determine dry mass. The specimens were divided in 10 groups of 10 specimens each. The groups were treated with different concentrations of PEG 400 (*M* ≈ 400 g/mol), PEG 1000 (*M* ≈ 1000 g/mol) (Carl Roth, Karlsruhe, Germany) and a PEG which provides methoxy- and trimethoxysilane functionality (Evonik, Essen, Germany) (Figure 1). The PEG-silane is of comparable chain length (*n* ≈ 7.5) and molecular mass (*M* ≈ 500 g/mol) to PEG 400 (*n* ≈ 8.5).

The specimens of each group were impregnated in a solution of 150 mL. The impregnation agent concentrations in demineralized water were varied by 15%, 30% and 45%. The reference specimens were impregnated with demineralized water only, to provide a comparable wetting and drying treatment and to determine eventual mass loss due to water-soluble extractives, which can be significant for oak wood. The impregnation was performed in a laboratory autoclave. In the first step, the pressure was reduced to 0.15 bar_abs_ for 30 min. In the second step, the pressure was raised up to 8 bar_abs_ for a duration of 12 h. Finally, the atmospheric pressure of 1 bar_abs_ was set up again. The specimens were dried at 103 °C for 48 h to determine the weight percent gain WPG ((dry-mass_treated_ − dry-mass_untreated_)/dry-mass_untreated_ × 100). The wood moisture content (WMC) is calculated as follows: (mass_moist_ − mass_dry_)/mass_dry_ × 100.

### 2.2. Determination of Anti-Shrinkage Efficiency (ASE)

Bulking (or swelling) is determined between two states of moisture content ((cross-section-swelling_moist_ − cross-section-swelling_dry_)/cross-section-swelling_dry_ × 100). To investigate the ASE ((cross-section-swelling_reference_ − cross-section-swelling_treated_)/cross-section-swelling_reference_ × 100), the following series of three typical indoor climatic conditions was investigated: 30 °C/70%relH, 20 °C/90%relH and 20 °C/95%relH. Full water saturation was determined during impregnation. Between the conditioning phases of 10 days, the specimens were dried at 103 °C for 48 h to determine potential leaching and dimensional changes. ASE calculation refers to untreated wood as reference. Since this reference is not the same specimen as the modified pendant, ASE is calculated via mean values and therefor no standard deviation is given. After the ASE investigation, half the samples were subjected to a leaching test according to EN 84 [33].

### 2.3. Scanning Electron Microscopy (SEM)

SEM was carried out on cross-sections of small PEG-silane impregnated specimen, using a Quanta™ 250 FEG (FEG-ESEM) (FEI, Hillsboro, OR, USA) device. Specimen of approximately 24 × 10 mm^2^ (on cross-section) were prepared and 1 mm of the cross-section surface was removed. Cross-sections were fresh cut using a razor blade and the measurements were taken under low vacuum at 60 Pa_abs_. Subsequently EDX (energy-dispersive X-ray spectroscopy) (Ametek materials Analytics Division, Berwyn, PA, USA) measurements were performed on the same specimens to investigate PEG-silane distribution.

### 2.4. Statistical Analysis

Statistical analysis was performed using PASW Statistics 18 software (version 18.0.0, IBM, New York, NY, USA). Analysis of variance (one-way ANOVA) was carried out, followed by a Post-Hoc Test according to the Scheffé procedure on a significance level of 0.05.

## 3. Results and Discussion

### 3.1. Effect of Impregnation on Specimen Mass and Water Relations

**Figure 2:**
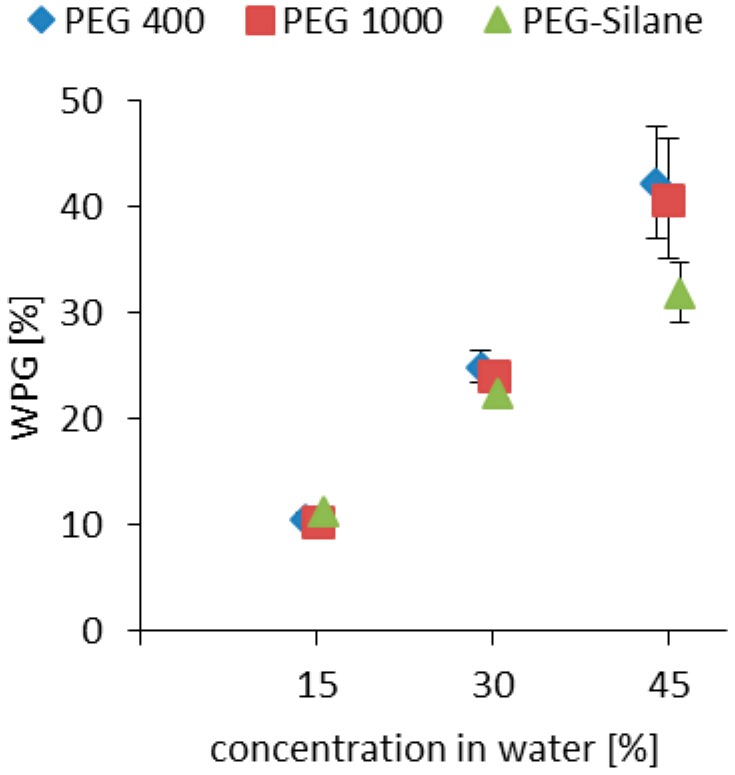
Weight percent gain (WPG) of impregnated oak wood achieved with different PEG impregnation agents and different concentrations of impregnation agent in water. Standard deviation given in whiskers. Different modification agent concentrations result in statistically significant different WPG.

The increase in specimen mass after impregnation expressed in terms of weight percent gain (WPG) is a widely used indicator of impregnation efficiency. The results of the WPG measurements confirm the suitability of a short-term vacuum-pressure impregnation method for PEG and PEG-silane in principle, especially in contrast to the classical impregnation method driven by diffusion only. The solution uptake upon impregnation for all variants was between 94% and 104%. It clearly can be seen that the impregnation agent concentration has a strong influence on the loading (WPG) of the samples (Figure 2). For PEG, irrespective of the molecular weight variant used, the lower concentration of 15% consistently resulted in WPG of 10–11%, whereas the higher concentration of 45% PEG led to up to 42% loading. PEG-silane, in contrast, did not achieve comparably high loadings. As shown in Figure 1, this PEG variant disposes of four methoxy functions for each molecule, which reduces overall polarity of the macromolecule and thus may be detrimental to its uptake by the polar cell wall. Overall, the loadings with impregnation agents are comparable to values from literature. Bjurhager et al. [26] impregnated oak wood samples with PEG 600 using a long term unpressurised method and achieved WPG values of up to 40%. Schneider [21] also found similar results for PEG-impregnated pine wood with WPG of up to 55% and 50% for beech wood. However, for good ASE, a high WPG is prerequisite but not sufficient by its own, because swelling after impregnation is a more relevant parameter.

**Figure 3:**
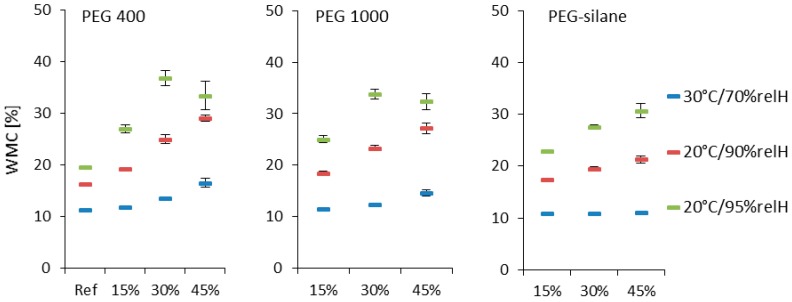
Wood moisture content (WMC) for different loaded oak wood in different climate conditions. Standard deviation given in whiskers. Different modification agent concentrations result in mostly statistically significant different WMC. Not significantly different are 15% concentrated PEG 400 and PEG 1000 compared to the reference, as well as 30% concentrated PEG silane to 15% and 45% concentrated PEG silane in 30 °C/70%relH.

Wood is capable of adsorbing humidity from the atmosphere until equilibrium moisture content is achieved. Roughly, this equilibrium moisture content may vary between 0% and 30% upon equilibration to environments with 0%relH and 100%relH, respectively. Variable density and extractive content of wood species may cause significant deviation from this rule of thumb. Modifications of the wood cell wall significantly diminish the ability of wood polymers to adsorb water either due to reduced availability of sorption sites in the case of active modification treatments, or due to steric hindrance caused by the presence of impregnation medium in the case of passive modification. Due to its distinct hydrophilicity PEG is different in this regard, as it contributes to the overall capacity of wood to adsorb water from humid environment. In extreme environments of ≥90%relH, PEG is able to adsorb up to 100% of its mass in water [17,34]. Therefore, the measured wood moisture content of the PEG-treated oak wood samples increases beyond the corresponding wood moisture level of the untreated reference (Figure 3). The same observation was made for PEG 1000 treated spruce [17], beech [21] and also oak [26]. Even if the increased moisture content of PEG-impregnated wood does not translate into increased swelling, which is limited by wood structure, water adsorbed to PEG may lead to an unpleasantly moist feeling of the wood surface [21]. In addition to this disadvantage, PEG may liquefy due to large amounts of water adsorbed and exude from treated wood in high-humidity environment. For example, when conditioned at 20 °C/95%relH, the mass of both, PEG_400_45% and PEG_1000_45% samples, diminished by approx. 10% due to exuding PEG.

**Figure 4:**
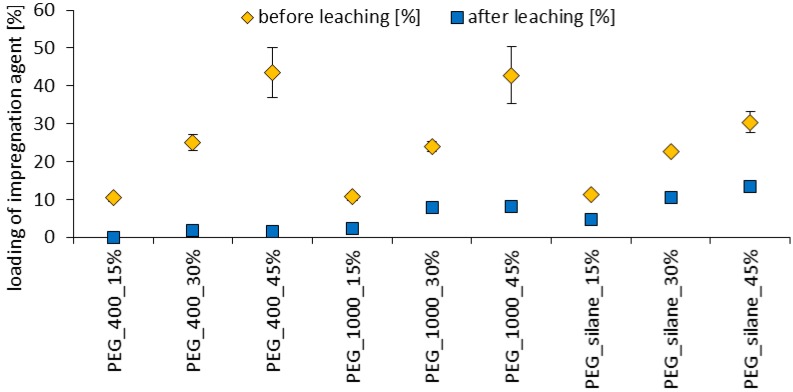
Impregnation agent loading before and after EN 84 [33] leaching test. Standard deviation given in whiskers. There is a significant loss for all samples.

PEG-silane, which differs in chemical structure from pure PEG, does not deviate from this pattern of wood-water relations in the gas-phase as shown in Figure 3. In the liquid phase, PEG is even more vulnerable to water due to its inherent water solubility (Figure 4). Corresponding to its excellent solubility in water, leaching of PEG 400 is almost complete after 14 days immersion in water (99% mean loss). For PEG 1000, less mass loss of 75% in mean due to leaching was observed during the same period of time. In clear contrast, PEG-silane was significantly more stable, even though a mass reduction of 56% still represents a very significant loss in impregnation agent. It is theoretically possible that this beneficial effect of silane functionality is due to auto-cross linking of PEG-silane, or event caused by attachment of silane functions to wood polymers [13], but this remains subject to speculation in the frame of the present study.

### 3.2. Dimensional Stabilization

**Figure 5:**
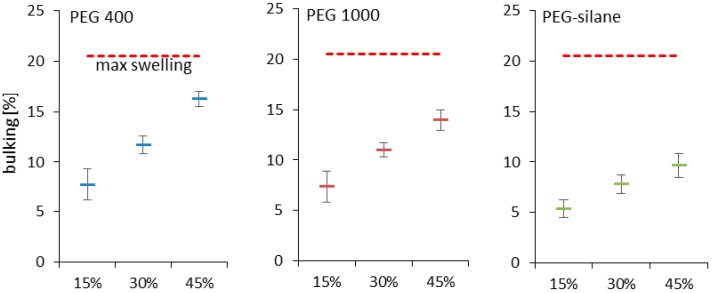
Transversal bulking of oak wood samples with different loadings, dry after impregnation. Maximum swelling of untreated oak wood displayed as dotted line. Standard deviation given in whiskers. Different modification agent concentrations result in significantly different bulking values.

Dimensional stabilization is the main target of PEG modification. Since swelling is very small in longitudinal wood direction, it is disregarded here. In a first step, overall effects of PEG impregnation on dimensional stability of oak wood will be discussed in terms of transverse swelling/shrinkage, without differentiating between the tangential and radial wood anatomical directions. Due to diffusion of PEG into the cell wall and replacement of water, the swollen state of the wood cell wall is partly preserved in the swollen state after drying, which is referred to as “bulking”. As shown in Figure 5, untreated oak wood is able to swell a maximum of 21% under water saturation (wood moisture content around 100%). After drying, untreated oak wood restores its former dimensions. When oak is treated with PEG-water solution, a certain amount auf PEG diffuses into the cell wall and stays there after drying-resulting in bulked dry-state of the wood. A bulking of 21% of oak wood after drying would entirely equalize dimension change by water. The difference between the maximum swelling minus bulking is the remaining swelling potential (Figure 5). In good agreement with the fact that increasing concentrations of impregnation medium consistently resulted in higher PEG-loadings in impregnated wood (Figure 2), increased bulking is observed with increasing concentrations of impregnation medium for all variant studied (Figure 5). Same as in terms of WPG, highest bulking was observed for PEG 400, followed by PEG 1000, and finally PEG-silane. Jeremic, et al. [30] found bulking values for PEG 1000 (30/70 PEG/water) impregnated pine wood of 16%, independent on the wood moisture content before the impregnation. These values are confirmed by the present results (Figure 5).

**Figure 6:**
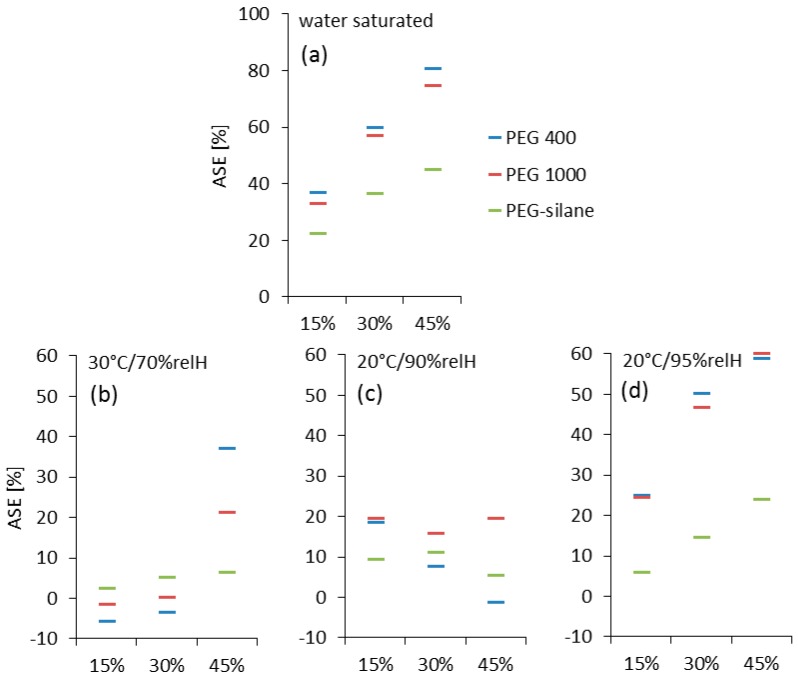
ASE values of treated oak wood in water saturated state (**a**) and different climate conditions (**b**–**d**), based on transversal swelling. The climate conditions in (**b**,**c**) correspond to approximately 11%, 16% and 20% wood equilibrium moisture content.

Bulking is a simple indicator of modification efficiency, as it gives a value for the remaining maximum range of dimensional instability. The anti-shrink efficiency (ASE) value determined in a series of equilibration experiments at different climates gives more insight into the effective reduction in shrinkage after PEG impregnation. The most common approach to determining ASE found in wood modification literature [3,12], is shrinkage to zero moisture from the fully water saturated state (Figure 6a), which is essentially the same information as expressed by the parameter bulking shown in Figure 5. High PEG loadings result in ASE values of up to 81%, while lower amounts still provide ASE values of 33% and more. For PEG-silane, smaller ASE values of 22% to 45% were achieved. While the pattern of ASE-dependence on impregnation agent loading is straightforward and clear when determined by drying to zero moisture from the fully swollen state (Figure 6a), ASE calculated from the dimensional changes measured during repeated equilibration experiments at varying climates shown in Figure 6b,c expose more complex relationships.

In the first, relatively dry cycle at 30 °C and 70%relH, specimens with low or intermediate PEG loading showed no significant ASE, whereas samples with high loading showed significant stabilization (Figure 6b). With 20 °C and 90%relH the second conditioning phase was more humid. In this regime, nearly all variants showed significant ASE between of 10–20% (Figure 6c). The final conditioning phase with 20 °C and 95%relH was the most humid one. Here, clear and systematic trends were observed (Figure 6d). Same as with bulking, a clear increase in ASE with increasing loading of impregnation agent is evident. Furthermore, PEG 400 and PEG 1000 perform significantly better that PEG-silane, again in good agreement with impregnation agent loadings. It is proposed that due to the comparably small changes in humidity and ensuing small changes in specimen dimensions occurring during the first two climate cycles, measurement inaccuracies may have contributed to unclear results in Figure 6b,c. By contrast changes observed with the most pronounced humid climate in cycle three (Figure 6d) were of sufficient magnitude in order to deliver reliable results in agreement with the outcome of other characterization experiments.

Until now, dimensional changes were discussed only in “transverse” direction, without discerning between the radial and the tangential wood anatomical directions. Analysis of swelling data resolved along these two directions may help to shed more light on potential mechanisms acting during PEG impregnation.

**Figure 7:**
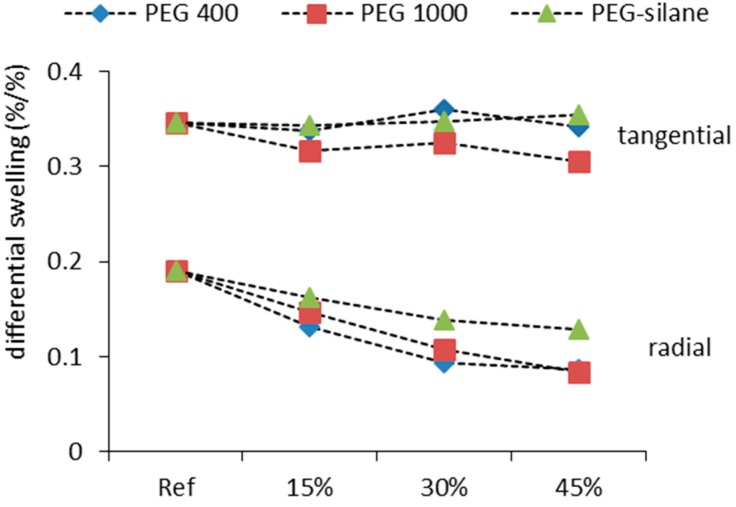
Differential swelling (percentage swelling per percentage change in moisture content) for differently treated oak wood specimens in radial and tangential anatomical directions (standard deviation of approx. 0.01 for radial and 0.02–0.03 for tangential direction not shown for better readability).

**Figure 8:**
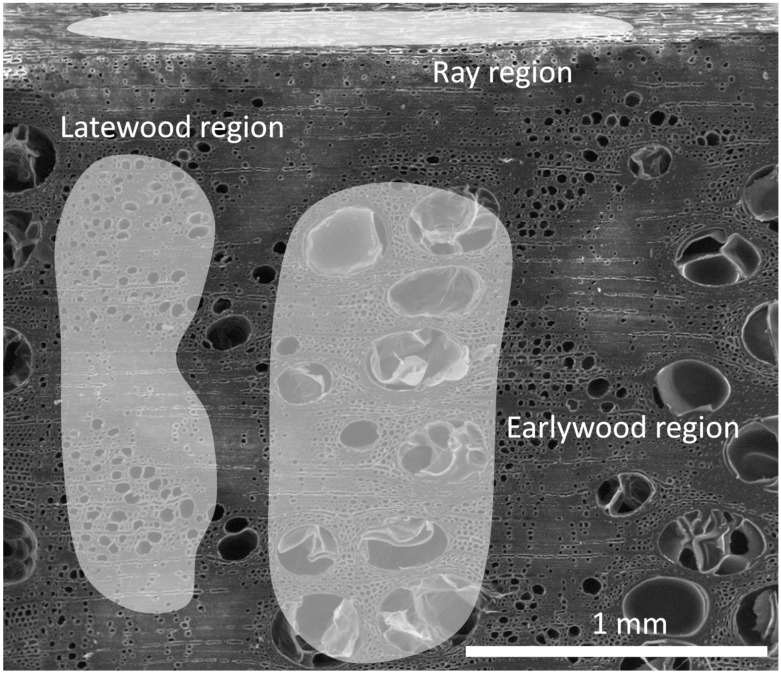
EDX area-measurements in the areas wood ray, late wood and early wood of a PEG-silane treated specimen.

As shown in Figure 7, resolving swelling along anatomical directions confirms macroscopic findings only for the radial direction, where essentially the same pattern as already reported for bulking (Figure 5) and ASE (Figure 6) is evident. Surprisingly, there is almost no effect, let alone a statistically significant effect, of treatment on tangential swelling. Thus, all effects of PEG impregnation on dimensional stability of oak wood observed in the present study are essentially due to modifications of radial swelling only, which is remarkable, even though similar trends were already reported [21]. An SEM study with PEG-silane, which has the advantage over PEG 400 and PEG 1000 of being detectable with EDX, provides some clues with regard to the cause of this surprising finding. It was revealed higher amounts of the treatment agent are located in early wood, compared to latewood, while no Si was detected in wood rays (Figure 8, Table 1). Wood rays are present in significant amount of up to 19.4% in oak wood tissue [35]. Due to its specific cell orientation, ray tissue has a stabilizing effect on radial swelling, whereas it contributes to tangential swelling in the same manner as surrounding fiber tissue. The fact that ray tissue is apparently unmodified, which is inferred from the fact that no Si is detected, it fully contributes to tangential swelling even in highly impregnated wood. Furthermore, when analyzing the different content of Si in early- and latewood (Table 1), the different densities of these tree ring regions have to be considered. Typically, oak earlywood has a density of 600 kg·m^−3^, whereas latewood density is around 800 kg·m^−3^ [36]. This difference in density exacerbates the differences in Si content revealed by EDX, which are based on an area concentration. By converting area concentration to mass concentration using the typical wood densities cited above, the at % Si in earlywood is roughly 4.4%, whereas it is only 2.5% in latewood. It is assumed that due to the presence of large-diameter vessels in earlywood, which provide pathways for flow of impregnation medium, earlywood is more efficiently impregnated and thus exhibits better ASE than latewood. Along the radial anatomical direction, earlywood and latewood layers alternate (serial configuration), and any dimensional change may be simply understood as the sum of dimensional changes of earlywood and latewood. Therefore, reduced earlywood shrinkage directly translates into reduced overall shrinkage in this anatomical direction. Contrarily, along the tangential direction earlywood and latewood are arranged in parallel configuration. In this setting latewood, which on average takes up 2/3 of an annual ring in the samples studied, is dominating the overall shrinkage behavior due to its comparably high width and density, which endows it with a comparably high elastic modulus.

## 4. Conclusions

The present work demonstrates the potential and limitations of PEG-impregnated oak wood for improved dimensional stability in indoor application. The key results can be summarized as follows:

- Beside the classical long-term diffusion approach, fast vacuum-pressure impregnation is suitable for PEG impregnation of European oak wood, as shown earlier for red pine. Significant reductions in swelling upon moisture uptake were achieved compared to untreated specimens, even though PEG impregnation resulted in above-reference equilibrium moisture content.
- Silane-functional PEG generally showed poorer performance compared to unmodified PEG, with the exception of leaching, where PEG-silane proved more recalcitrant than PEG and therefore demands deeper attention in further work to overcome unmodified PEG disadvantages.
- Dimensional stabilization was predominantly achieved in oak earlywood, which resulted in dimensional stabilization only in radial anatomical direction, whereas the tangential dimensional stability remained unaffected by impregnation.
- The suitability of the method for the bigger scale specimens needs to be proven in future as the next step towards industrial application.

## Figures and Tables

**Table 1:** Results of EDX area-measurements, as shown in Figure 8.

Region	Element	at %
Wood ray	C	59.46
	O	40.54
	Si	-
Late wood	C	59.42
	O	38.59
	Si	1.99
Early wood	C	60.65
	O	36.70
	Si	2.65
